# The complete chloroplast genome sequences of three *Cypripedium* species and their phylogenetic analysis

**DOI:** 10.1038/s41598-025-98287-3

**Published:** 2025-04-18

**Authors:** Qun Wang, Jing An, Yan Wang, Baoqiang Zheng

**Affiliations:** 1 Key Laboratory of Tree Breeding and Cultivation of National Forestry and Grassland Administration, Research Institute of Forestry, Chinese Academy of Forestry, Beijing, 100091 China; 2 Beijing Songshan Natural Reserve Administration, Beijing, 102100 China

**Keywords:** *Cypripedium Macranthos* Swartz, *Cypripedium* × *ventricosum* Swartz, *Cypripedium shanxiense* S. C. Chen, Chloroplast genome (cpDNA), Phylogeny, Evolution, Molecular biology

## Abstract

*Cypripedium macranthos* Swartz, *C.* × *ventricosum* Swartz, and *C. shanxiense* S. C. Chen were highly promising ornamental plants. In this study, the latest complete chloroplast genome sequence of *C. macranthos*, *C.* × *ventricosum*, and *C. shanxiense* were reported using correct sample material from their native range, and their phylogenetic relationships with other related species were investigated preliminarily. The whole chloroplast genome lengths of *C. macranthos*, *C.* × *ventricosum*, and *C. shanxiense* were 181,030 bp, 175,385 bp, and 177,627 bp, respectively, with total GC contents of 34.56%, 34.48%, and 34.42%, respectively. Based on the maximum likelihood phylogenetic tree from the chloroplast genome sequences and internal transcribed spacer (ITS) sequences, it was confirmed that *C.* × *ventricosum* is most closely related to *C. calceolus*, and the idea that *C.* × *ventricosum* is an interspecific hybrid between *C. calceolus* and *C. macranthos* is supported. By integrating the results of phylogenetic analysis, genomic structural comparison, and considerations of sampling locations, it is evident that the former chloroplast genome of *C. macranthos* is inaccurate. This study provides crucial information for research on the phylogeny, genetics, and conservation of *C. macranthos*, *C.* × *ventricosum*, and *C. shanxiense*.

**Supplementary Information:**

The online version contains supplementary material available at 10.1038/s41598-025-98287-3.

## Introduction

**Fig. 1:**
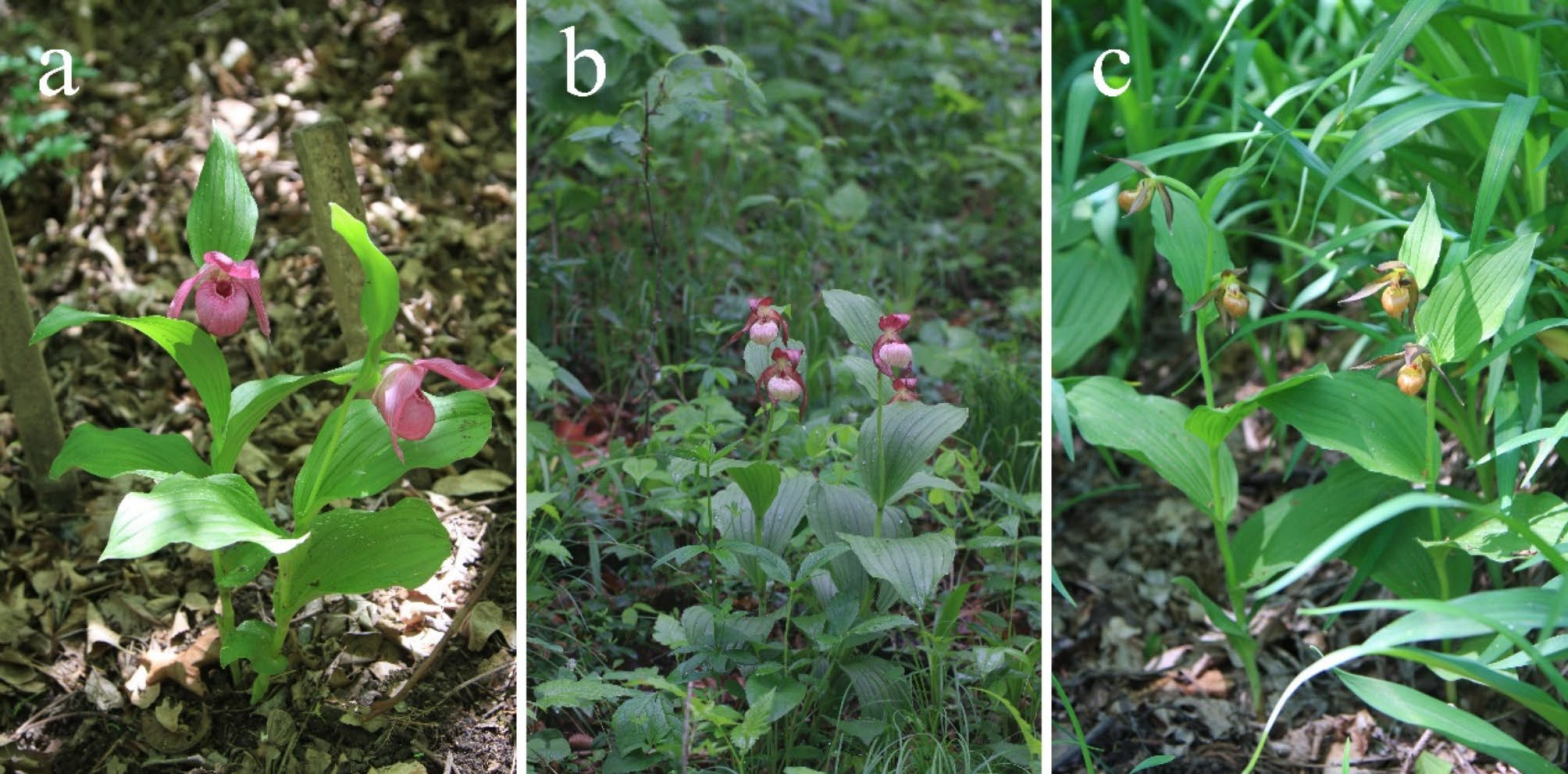
Reference image of *C. macranthos*, *C.* × *ventricosum*, and *C. shanxiense* flowering plant. (**a**) Reference image of *C. macranthos*; (**b**) Reference image of *C.* × *ventricosum*; (**c**) Reference image of *C. shanxiense*. All of the above images were taken by Mr. Baoqiang Zheng. Images a and b were photographed in Sandawan Township, Yanji County, Jilin Province, China (43.2°N, 129.1°E), and image c was taken in the Beijing Songshan Nature Reserve (40.3°N, 115.4°E). All images are unpublished (used with permission).

The genus *Cypripedium*, a subdivision within the Orchidaceae family, is renowned for its species that feature a labellum evolved into a slipper-like structure, commonly known as slipper orchids or lady’s slippers^1,2^. *Cypripedium macranthos* Swartz (1800) (Fig. 1a) and *C.* × *ventricosum* Swartz (1800) (Fig. 1b) are two such species that thrive in cooler climates and are not tolerant of high temperatures. They are among the few orchids that inhabit temperate, high-latitude regions and have a global distribution limited to Russia, China, Japan, and the Korean Peninsula^2–5^. Their distinctive petal shapes and vivid bloom colors endow *C. macranthos* and *C.* × *ventricosum* with significant ornamental value, ranking them among the most favored orchids^4^. *C. shanxiense* S. C. Chen (1983) (Fig. 1c) is mainly found in northern and western China, northern Japan, southeastern Russia, and across Mongolia^5,6^. Distinct from most *Cypripedium* species, *C. shanxiense* boasts small, delicate flowers with unique colors that are neither purely greenish-yellow nor yellow, nor bright crimson or purplish-red, yet it retains a high ornamental value and shows promise for garden landscape applications.

Successful hybridization between genera and species of Orchidaceae plants often exists^6^. *C.* × *ventricosum* often grows in association with *C. calceolus* and *C. macranthos*, and it is therefore considered to be a natural hybrid of these two species^5–7^. However, there is still insufficient molecular evidence to confirm their relationship. In the past decade, habitat loss, biological factors (such as regeneration challenges) and over-collection of these beautiful wildflowers for horticultural and medicinal purposes have brought these wild species dangerously close to extinction^3,4,8^. To date, few studies have been conducted on these beautiful and endangered orchids.

The chloroplast genome is a valuable source of information for studying plant phylogeny and evolution, which is attributed to its predominantly maternal inheritance^9–12^. With the advancement of sequencing technologies, an increasing number of plant chloroplast genomes are being discovered, which contributes to our understanding of plant phylogeny^13,14^. While Luo et al. have published the chloroplast genome of *C. macranthos*, the sample collection site in Yunnan, China, characterized by a tropical or subtropical monsoon climate, does not align with the cool-temperate habitat preferences of *C. macranthos*^15^. Consequently, we have sampled from the native distributions of *C. macranthos*, *C.* × *ventricosum*, and *C. shanxiense*, and in this paper, we report the most up-to-date chloroplast genomes for these species, along with phylogenetic analyses. Our findings are poised to contribute valuable insights for future research, development, and application related to *C. macranthos*, *C.* × *ventricosum*, and *C. shanxiense*.

## Results

### Assembly and annotation of the Chloroplast genome

**Fig. 2:**
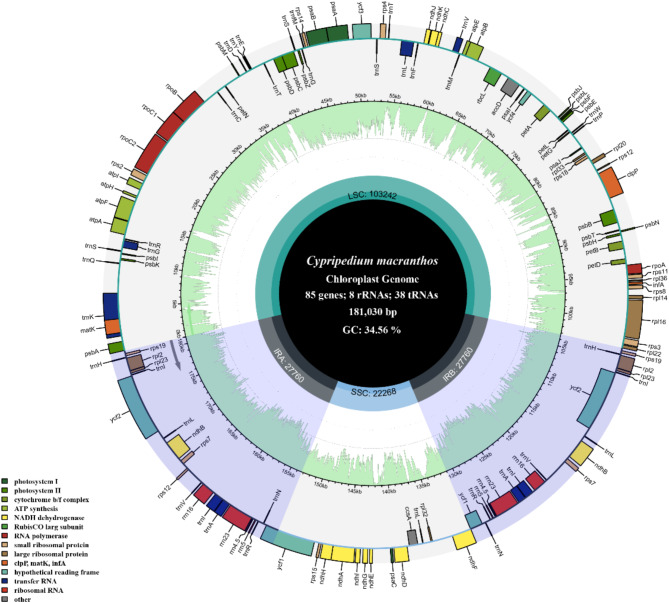
Chloroplast genome map of *C. macranthos*. Genes belonging to different functional groups are shown in different colors. Genes within the circle undergo clockwise transcription, while those outside the circle undergo counterclockwise transcription. The functional classification of the genes is shown in the bottom left corner. The light green inner circle indicates the GC content of the chloroplast genome.

The entire chloroplast genome of *C. macranthos* was a circular molecule composed of 181,030 bp, comprising four contiguous segments: a large single-copy region (LSC) of 103,242 bp, a short single-copy region (SSC) of 22,268 bp, and two inverted repeat regions (IRA and IRB) of 27,760 bp each, with a total GC content of 34.56% (Fig. 2). The average sequencing depth was 543 × (Figure S7). The genome contained 131 genes, including 85 protein-coding genes, 38 transfer RNA (tRNA) genes, and 8 ribosomal RNA (rRNA) genes. Among these, 8 protein-coding genes (*atpF*, *rpoC1*, *petB*, *petD*, *rpl16*, *rpl2*, *ndhB*, *ndhA*) and 6 tRNA genes (*trnK-UUU*, *trnG-UCC*, *trnL-UAA*, *trnV-UAC*, *trnI-GAU*, *trnA-UGC*) contained one intron, 2 protein-coding genes (*ycf3*, *clpP*) contained two introns (Figure S1), and 1 gene contained a trans-splicing gene (*rps12*) (Figure S2).

**Fig. 3:**
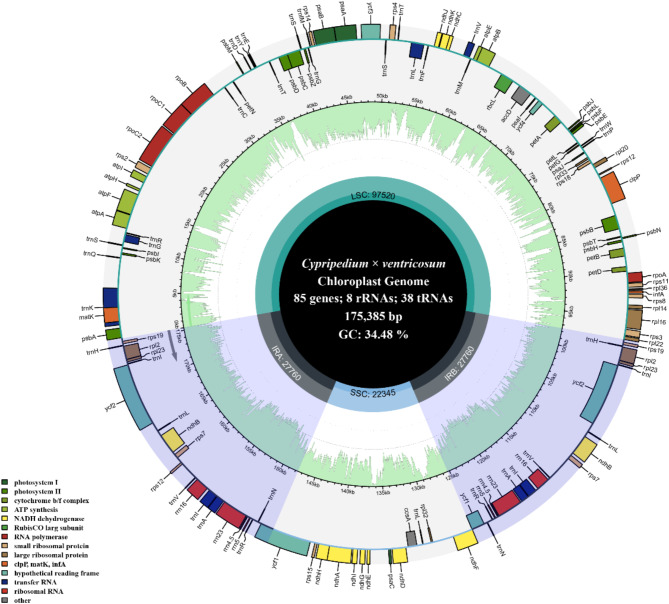
Chloroplast genome map of *C.* × *ventricosum*. Genes belonging to different functional groups are shown in different colors. Genes within the circle undergo clockwise transcription, while those outside the circle undergo counterclockwise transcription. The functional classification of the genes is shown in the bottom left corner. The light green inner circle indicates the GC content of the chloroplast genome.

The chloroplast genome of *C.* × *ventricosum* was a circular molecule totaling 175,385 bp, with a GC content of 34.48%. It consisted of four contiguous segments: two inverted repeat regions (IRA and IRB) of 27,760 bp each, a large single-copy region (LSC) of 97,520 bp, and a short single-copy region (SSC) of 22,345 bp (Fig. 3). The average sequencing depth was 282 × (Figure S8). The genome contained a total of 131 genes, including 85 protein-coding genes, 38 tRNA genes, and 8 rRNA genes. Among these, 8 protein-coding genes (*atpF*, *rpoC1*, *petB*, *petD*, *rpl16*, *rpl2*, *ndhB*, *ndhA*) and 6 tRNA genes (*trnK-UUU*, *trnG-UCC*, *trnL-UAA*, *trnV-UAC*, *trnI-GAU*, *trnA-UGC*) contained one intron, 2 protein-coding genes (*ycf3*, *clpP*) contained two introns (Figure S3), and 1 gene contained a trans-spliced gene (*rps12*) (Figure S4).

**Fig. 4:**
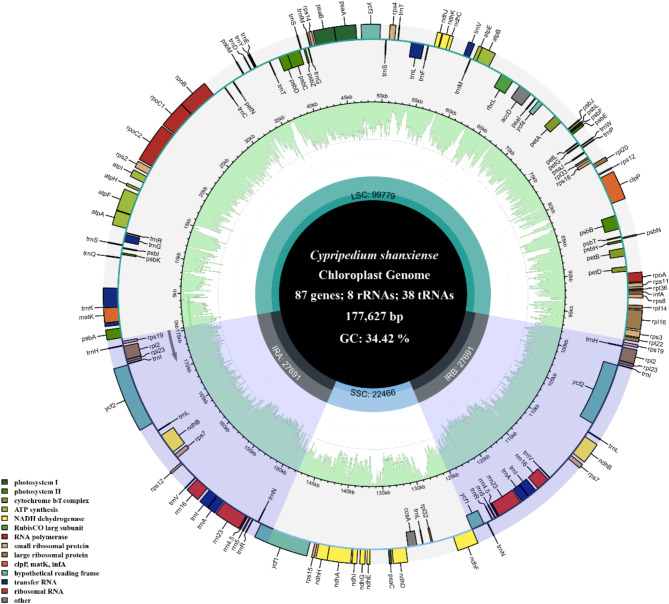
Chloroplast genome map of *C. shanxiense*. Genes belonging to different functional groups are shown in different colors. Genes within the circle undergo clockwise transcription, while those outside the circle undergo counterclockwise transcription. The functional classification of the genes is shown in the bottom left corner. The light green inner circle indicates the GC content of the chloroplast genome.

The chloroplast genome of *C. shanxiense* was a circular molecule of 177,627 bp, composed of four continuous segments: two inverted repeat regions (IRA and IRB), each 27,691 bp; a large single-copy region (LSC), 99,779 bp; and a short single-copy region (SSC), 22,466 bp, with a total GC content of 34.42% (Fig. 4). The average sequence depth was 420 × (Figure S9). A total of 133 genes were identified, including 87 protein-coding genes, 38 tRNA genes, and 8 rRNA genes. Among these, 9 protein-coding genes (*rps16*, *atpF*, *rpoC1*, *petB*, *petD*, *rpl16*, *rpl2*, *ndhB*, *ndhA*) and 6 tRNA genes (*trnK-UUU*, *trnG-UCC*, *trnL-UAA*, *trnV-UAC*, *trnI-GAU*, *trnA-UGC*) contained one intron, 2 protein-coding genes (*ycf3*, *clpP*) contained two introns (Figure S5), and 1 gene (*rps12*) contained a trans-spliced intron (Figure S6).

## Phylogenetic analysis

**Fig. 5:**
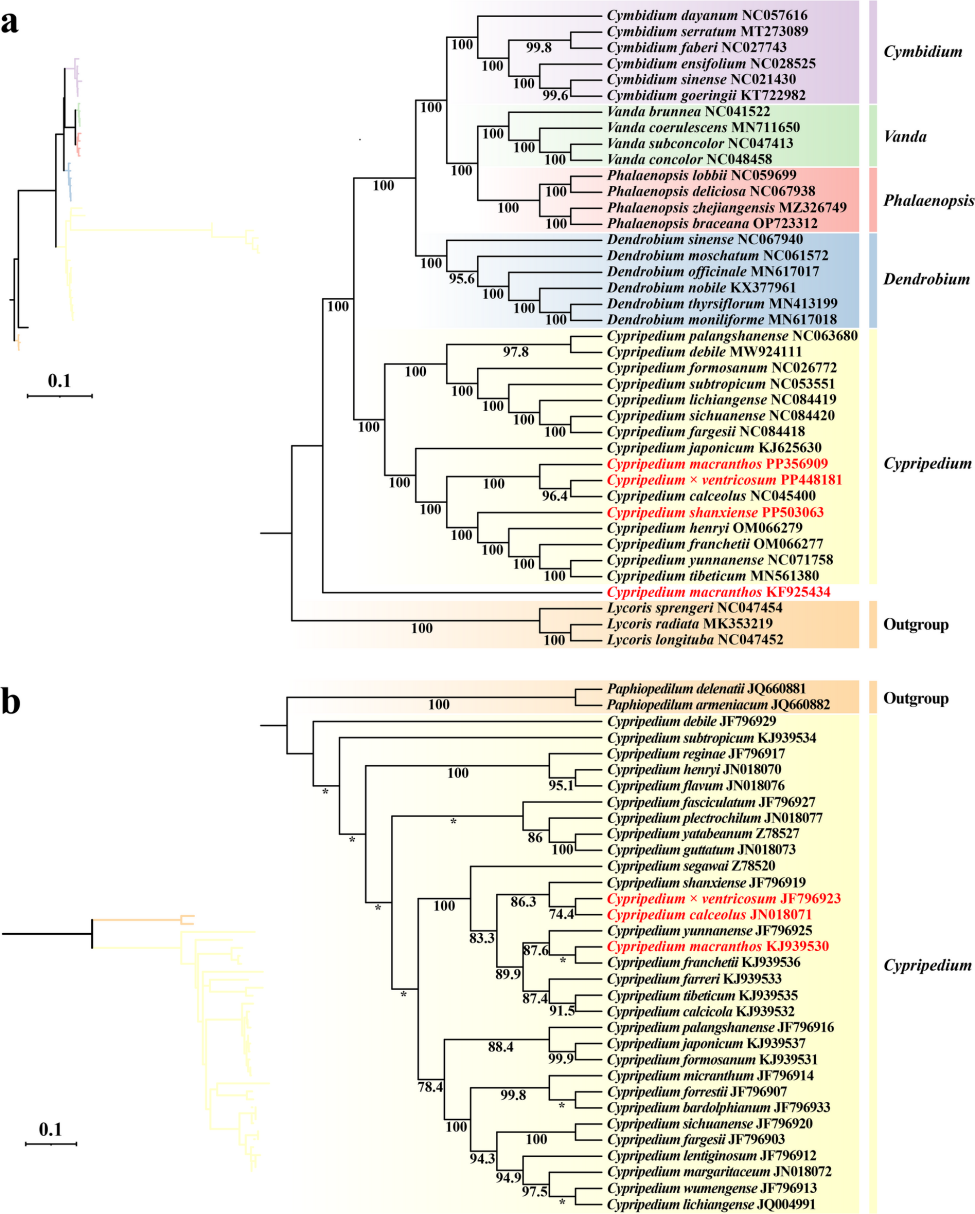
Chloroplast genome and ITS phylogenetic trees. ML tree based on the whole chloroplast genome of *C. macranthos*, *C.* × *ventricosum*, *C. shanxiense*, and 34 Orchidaceae species, with 3 Amaryllidaceae species as outgroups (**a**). ML tree based on ITS sequences from 31 *Cypripedium* species, with 2 *Paphiopedilum* species as outgroups (**b**). The numbers at each node represent the bootstrap values from 1000 repetitions. “*” indicates that the bootstrap value is below 70. On the left side of each tree, a ML tree that preserves genetic distances is displayed, with branch colors corresponding to the genus-specific background colors indicated in the figure on the right.

A phylogenetic tree can be represented by a branching diagram that illustrates the relationships between similar organisms in a tree-like structure^16^. The chloroplast genome sequences of 34 Lythraceae species and 3 Amaryllidaceae species were selected from NCBI to investigate the phylogeny of *C. macranthos*, *C.* × *ventricosum*, and *C. shanxiense* (Fig. 5a). According to the phylogenetic tree, species from the same genus were grouped together in one branch, while species from different genera diverged. Each node had excellent support (BS ≥ 95.6). However, the former *C. macranthos* (submitted by Luo et al.^15^) was not placed in the same branch as the other *Cypripedium* species. The latest chloroplast genome of *C. macranthos* (submitted by us) was most closely related to *C. calceolus* and *C.* × *ventricosum*, and *C.* × *ventricosum* was positioned between the branches of *C. calceolus* and *C. macranthos*.

To further assess the relationship between *C.* × *ventricosum* and *C. macranthos* as well as *C. calceolus*, a phylogenetic analysis was conducted on the internal transcribed spacer (ITS) sequences of 31 *Cypripedium* species and 2 *Paphiopedilum* species. Based on the branching pattern of the tree, *C.* × *ventricosum* was most closely related to *C. calceolus*, and it is positioned between the branches of *C. calculous* and *C. macranthos*, which also suggests that it might be a hybrid descendant of these two species (Fig. 5b).

## Genome structure analysis

**Fig. 6:**
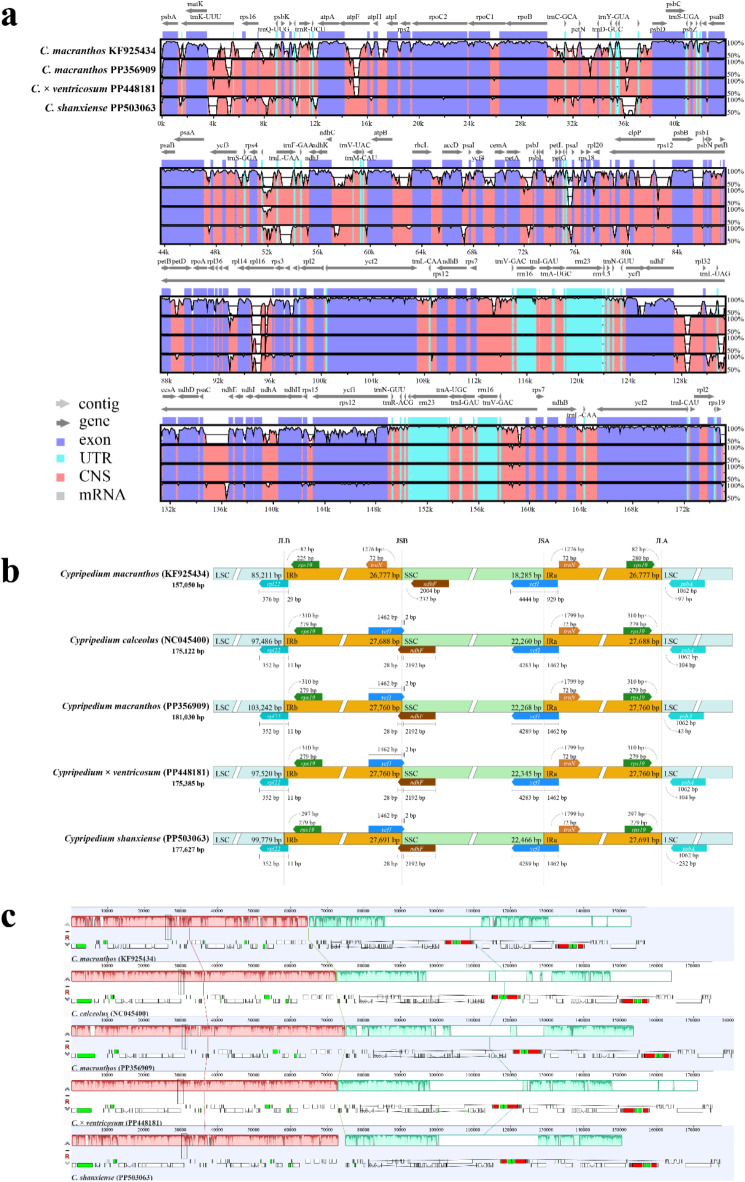
Comparison of chloroplast genome structures in five *Cypripedium* species. Visualization of chloroplast genome sequence alignment for four *Cypripedium* species using *C. calceolus* as a reference (**a**). The vertical scale shows the percent of identity, ranging from 50 to 100%. The horizontal axis shows the coordinates within the chloroplast genome. Comparison of the LSC, SSC, and IR boundaries among five *Cypripedium* chloroplast genomes (**b**). The LSC, SSC, and IR regions are shown in different colors. JLB, JSB, JSA, and JLA represent the junction sites between the corresponding regions of the genome, respectively. Genes are depicted as boxes. Collinearity analysis of the chloroplast genomes from five *Cypripedium* species (**c**).

Based on the chloroplast genome sequence alignment results, it can be observed that the latest *C. macranthos*, *C.* × *ventricosum*, and *C. shanxiense* exhibit high conservation, with variations in the non-coding regions being higher than those in the coding regions. Most of the high mutation areas are also located within the conserved non-coding sequences (CNS). In contrast, the former *C. macranthos* also exhibits significant variation in the exon regions (Fig. 6a). The comparison of the IR boundaries among *C. calceolus*, the former *C. macranthos*, the latest *C. macranthos*, *C.* × *ventricosum*, and *C. shanxiense* reveals that *C. calceolus*, the latest *C. macranthos*, *C.* × *ventricosum*, and *C. shanxiense* are relatively conservative at the IR boundaries. The main difference of the former *C. macranthos* from the other four is the absence of *ycf1* at the IRb-SSC boundary, and *ndhF* does not span the SSC-IRb boundary (Fig. 6b). Based on the collinearity analysis of the chloroplast genomes, it was found that *C. calceolus*, the former *C. macranthos*, the latest *C. macranthos*, *C.* × *ventricosum*, and *C. shanxiense* did not undergo rearrangements or inversions (Fig. 6c).

## Discussion

Chloroplast genomics is a research hotspot for the study of plant evolutionary relationships because the plant chloroplast genome has a comparatively stable and conservative structure and includes extensive genetic information, which is an important basis for the study of plant evolutionary relationships^17,18^. In this study, the latest complete chloroplast genome of *C. macranthos*, *C.* × *ventricosum*, and *C. shanxiense* were assembled and phylogenetically analyzed with the published chloroplast genomes of 34 species of Orchidaceae and 3 of Amaryllidaceae. The phylogenetic analysis revealed that distinct genera diverged from one another and that species belonging to the same genus were clustered into a single branch, indicating the presence of distinct boundaries between various genera. Interestingly, species of *Cypripedium* can be clearly distinguished into two groups within a single branch, suggesting a clear differentiation event in *Cypripedium*.

Previously, Li et al. cloned six genes and conducted a phylogenetic analysis of the *Cypripedium* genus, which did not include *C.* × *ventricosum*, but found that *C. calceolus* is most closely related to *C. shanxiense*, rather than *C. macranthos*^19^. In contrast, the phylogenetic analysis of the entire chloroplast genome in this study indicates a close relationship between *C.* × *ventricosum* and both *C. calceolus* and *C. macranthos*, with *C.* × *ventricosum* positioned between the branches of *C. calceolus* and *C. macranthos*. Meanwhile, *C. shanxiense* is most closely related to *C. henryi*. In addition, this study conducted a phylogenetic analysis based on the known ITS sequences of 31 *Cypripedium* species, and the results indicate that *C.* × *ventricosum* is most closely related to *C. calceolus*, and it is also positioned between the branches of *C. calceolus* and *C. macranthos*. These results indicate that the findings of this study support the view proposed by earlier scholars that *C.* × *ventricosum* is an inter-specific hybrid between *C. calceolus* and *C. macranthos*^5–7^. Additionally, the former *C. macranthos* did not cluster with other *Cypripedium* species in the same branch. Through structural comparative analysis of the genome, we found that the former *C. macranthos* chloroplast genome did not undergo rearrangements or inversions within the genome. This may suggest that the material from Luo et al. was not a pure strain of *C. macranthos*^15^. Given the natural hybridization of Orchidaceae species and the sampling location of Luo et al., it is speculated that this is likely a hybrid species that was mistakenly identified as *C. macranthos* due to its striking similarity to *C. macranthos*^6,15^.

In conclusion, this study supports the idea that *C.* × *ventricosum* is an interspecific hybrid between *C. calceolus* and *C. macranthos* from a molecular biology perspective. The reliable complete chloroplast genome of *C. macranthos*, *C.* × *ventricosum*, and *C. shanxiense* in this study will provide important theoretical support for the accurate and rapid identification of *C. macranthos*, *C.* × *ventricosum*, and *C. shanxiense* species, the scientific delineation of relatives, and the mechanism of genetic evolution, and will also be of great significance to the cultivation of high-quality germplasm resources and the conservation of wild resources of *C. macranthos*, *C.* × *ventricosum*, and *C. shanxiense*.

## Materials and methods

### Plant materials

Fresh leaves of *C. macranthos* and *C.* × *ventricosum* were collected from Sandaowan Town, Yanji County, Jilin Province, China (43.2°N; 129.1°E), and fresh leaves of *C. shanxiense* were collected from Songshan Nature Reserve, Beijing, China (40.3°N, 115.4°E). The specimens of *C. macranthos*, *C.* × *ventricosum*, and *C. shanxiense* were stored in the flower herbarium of the Research Institute of Forestry, Chinese Academy of Forestry, and can be accessed through the contact person Baoqiang Zheng (zhengbaoqiang@aliyun.com) under the registration number CM0008, CV0004 and CS0014, respectively.

## Genome sequencing, assembly, and annotation

Using the Fast Plant Genomic DNA Isolation Kit (Sangon Biotech Co., Ltd., Shanghai, China), total DNA was recovered. Hieff NGS^®^MaxUp II DNA Library Prep Kit for Illumina^®^ (YEASEN, Shanghai, China) was used for sequencing library construction. The Illumina Novaseq 6000 sequencing platform (Illumina, San Diego, USA) was used to generate paired-ended raw reads.

Fastp v0.36 was used as a tool for quality control of the sequencing data^20^. Sequencing depth and coverage were calculated using BEDTools v2.31.1 and mapping was performed using R-ggplot2^21^. Bowtie2 v2.1.0 was used to splice the sequenced segments^22^. The chloroplast genome was assembled using GetOrganelle v1.7.5.3 with *C. calceolus* (NC045400) as a reference^23,24^.

CPGAVAS2 (http://47.96.249.172:16019/analyzer/home) was used to annotate the chloroplast genome^25^. tRNAs were identified using tRNAscan-SE v2.0^26^. BLAST and DOGMA (http://dogma.ccbb.utexas.edu/) were used to verify the annotations^27,28^. The annotation of the chloroplast genome of *C. macranthos*, *C.* × *ventricosum*, and *C. shanxiense* were uploaded to GenBank (accession number: PP356909, PP448181, PP503063).

Circos (http://circos.ca/) was used to map circular genes^29^. CPGview (http://www.1kmpg.cn/cpgview/) was used to map cis- and trans-splicing genes^30^.

## Phylogenetic analysis

Phylogenetic trees were constructed using chloroplast genome sequences and ITS sequences, respectively. Sequence alignment was performed using MAFFT v7 (https://mafft.cbrc.jp/)^31^. The Gblocks module in PhyloSuite v1.2.2 was used for trimming, and the ModelFinder module was used to determine the best models for the chloroplast genome sequence phylogenetic tree and the ITS sequence phylogenetic tree, which were GTR + F + R4 and SYM + G4, respectively. The IQ-TREE module was used to perform maximum likelihood (ML) analysis^32^. The phylogenetic tree was visualized using iTOL v6 (https://itol.embl.de/)^33^. The chloroplast genome sequences and ITS sequences used for phylogenetic analysis were detailed in Tables 1 and 2, respectively.

**Table 1:** Chloroplast genome sequences for phylogenetic analysis.

Name	Accession number	Reference	Name	Accession number	Reference
*Cypripedium*			*Dendrobium*		
*C. macranthos*	PP356909	This study	*D. nobile*	KX377961	Xue et al.^34^
*C. macranthos*	KF925434	Luo et al.^15^	*D. moniliforme*	MN617018	Kim et al.^35^
*C.* × *ventricosum*	PP448181	This study	*D. sinense*	NC067940	Unpublish
*C. shanxiense*	PP503063	This study	*D. officinale*	MN617017	Luo et al.^15^
*C. sichuanense*	NC084420	Unpublish	*D. moschatum*	NC061572	Yang et al.^36^
*C. henryi*	OM066279	Unpublish	*D. thyrsiflorum*	MN413199	Zhu et al.^37^
*C. franchetii*	OM066277	Unpublish	*Cymbidium*		
*C. lichiangense*	NC084419	Unpublish	*C. serratum*	MT273089	Shao and Ning^38^
*C. fargesii*	NC084418	Unpublish	*C. dayanum*	NC057616	Du et al.^39^
*C. yunnanense*	NC071758	Unpublish	*C. goeringii*	KT722982	Unpublish
*C. tibeticum*	MN561380	Li et al.^40^	*C. faberi*	NC027743	Unpublish
*C. subtropicum*	NC053551	Guo et al.^41^	*C. sinense*	NC021430	Yang et al.^42^
*C. calceolus*	NC045400	Zhang et al.^23^	*C. ensifolium*	NC028525	Unpublish
*C. palangshanense*	NC063680	Zhang et al.^43^	*Vanda*		
*C. debile*	MW924111	Zhang et al.^43^	*V. coerulescens*	MN711650	Wang et al.^44^
*C. japonicum*	KJ625630	Kim et al.^45^	*V. concolor*	NC048458	Liu et al.^46^
*C. formosanum*	NC026772	Lin et al.^47^	*V. subconcolor*	NC047413	Chen et al.^48^
*Phalaenopsis*			*V. brunnea*	NC041522	Li et al.^49^
*P. deliciosa*	NC067938	Unpublish	*Lycoris*		
*P. zhejiangensis*	MZ326749	Jiang et al.^50^	*L. sprengeri*	NC047454	Liu et al.^51^
*P. lobbii*	NC059699	Zhang et al.^52^	*L. longituba*	NC047452	Liu et al.^51^
*P. braceana*	OP723312	Unpublish	*L. radiata*	MK353219	Liu et al.^51^

**Table 2:** ITS sequences for phylogenetic analysis.

Name	Accession number	Reference	Name	Accession number	Reference
*Cypripedium*			*C. macranthos*	KJ939530	Szlachetko et al.^53^
*C. × ventricosum*	JF796923	Li et al.^19^	*C. margaritaceum*	JN018072	unpublish
*C. bardolphianum*	JF796933	Li et al.^19^	*C. micranthum*	JF796914	Li et al.^19^
*C. calceolus*	JN018071	Unpublish	*C. palangshanense*	JF796916	Li et al.^19^
*C. calcicola*	KJ939532	Szlachetko et al.^53^	*C. plectrochilum*	JN018077	Unpublish
*C. debile*	JF796929	Li et al.^19^	*C. reginae*	JF796917	Li et al.^19^
*C. fargesii*	JF796903	Li et al.^19^	*C. segawai*	Z78520	Unpublish
*C. farreri*	KJ939533	Szlachetko et al.^53^	*C. shanxiense*	JF796919	Li et al.^19^
*C. fasciculatum*	JF796927	Li et al.^19^	*C. sichuanense*	JF796920	Li et al.^19^
*C. flavum*	JN018076	Unpublish	*C. subtropicum*	KJ939534	Szlachetko et al.^53^
*C. formosanum*	KJ939531	Szlachetko et al.^53^	*C. tibeticum*	KJ939535	Szlachetko et al.^53^
*C. forrestii*	JF796907	Li et al.^19^	*C. wumengense*	JF796913	Li et al.^19^
*C. franchetii*	KJ939536	Szlachetko et al.^53^	*C. yatabeanum*	Z78527	Unpublish
*C. guttatum*	JN018073	Unpublish	*C. yunnanense*	JF796925	Li et al.^19^
*C. henryi*	JN018070	Unpublish	*Paphiopedilum*		
*C. japonicum*	KJ939537	Szlachetko et al.^53^	*P. armeniacum*	JQ660882	Gorniak et al.^54^
*C. lentiginosum*	JF796912	Li et al.^19^	*P. delenatii*	JQ660881	Gorniak et al.^54^
*C. lichiangense*	JQ004991	Unpublish			

### Comparative analysis of genome structure

Using *C. calceolus* (NC045400) as the reference genome, a visual comparison of the chloroplast genomes of the former *C. macranthos* (KF925434), the latest *C. macranthos* (PP356909), *C.* × *ventricosum* (PP448181), and *C. shanxiense* (PP503063) was conducted with mVISTA (https://genome.lbl.gov/vista/index.shtml), selecting the Shuffle-LAGAN mode^55^.

Using CPJSdraw (V1.0.0) to generate a visual map of the IR boundaries for the chloroplast genomes of *C. calceolus* (NC045400), the former *C. macranthos* (KF925434), the latest *C. macranthos* (PP356909), *C.* × *ventricosum* (PP448181), and *C. shanxiense* (PP503063)^56^.

Conducting a collinear analysis of the chloroplast genomes of *C. calceolus* (NC045400), the former *C. macranthos* (KF925434), the latest *C. macranthos* (PP356909), *C.* × *ventricosum* (PP448181), and *C. shanxiense* (PP503063) using Mauve (https://darlinglab.org/mauve/mauve.html).

## Electronic supplementary material

Below is the link to the electronic supplementary material.

Supplementary Figures

## Data Availability

The NCBI database in GenBank (https://www.ncbi.nlm.nih.gov/) has the *C. macranthos*, *C.* × *ventricosum*, and *C. shanxiense* genome sequence data supporting the findings of this study available to the public under the accession number PP356909, PP448181, and PP503063, respectively. The associated BioProject, BioSample and Sequence Read Archive numbers with *C. macranthos *are PRJNA1078557, SAMN40005752 and SRR28025806, respectively. The associated BioProject, BioSample and Sequence Read Archive numbers with *C. *× *ventricosum* are PRJNA1084227, SAMN40275977 and SRR28236982, respectively. The associated BioProject, BioSample and Sequence Read Archive numbers with *C. shanxiense* are PRJNA1088625, SAMN40479335, and SRR28362302, respectively.
