# Supplementary figures and images for: The complete chloroplast genome sequences of three Cypripedium species and their phylogenetic analysis
Source: Sci Rep. 2025 Apr 18;15:13461. doi: 10.1038/s41598-025-98287-3 (PMC12008232; doi:10.1038/s41598-025-98287-3)

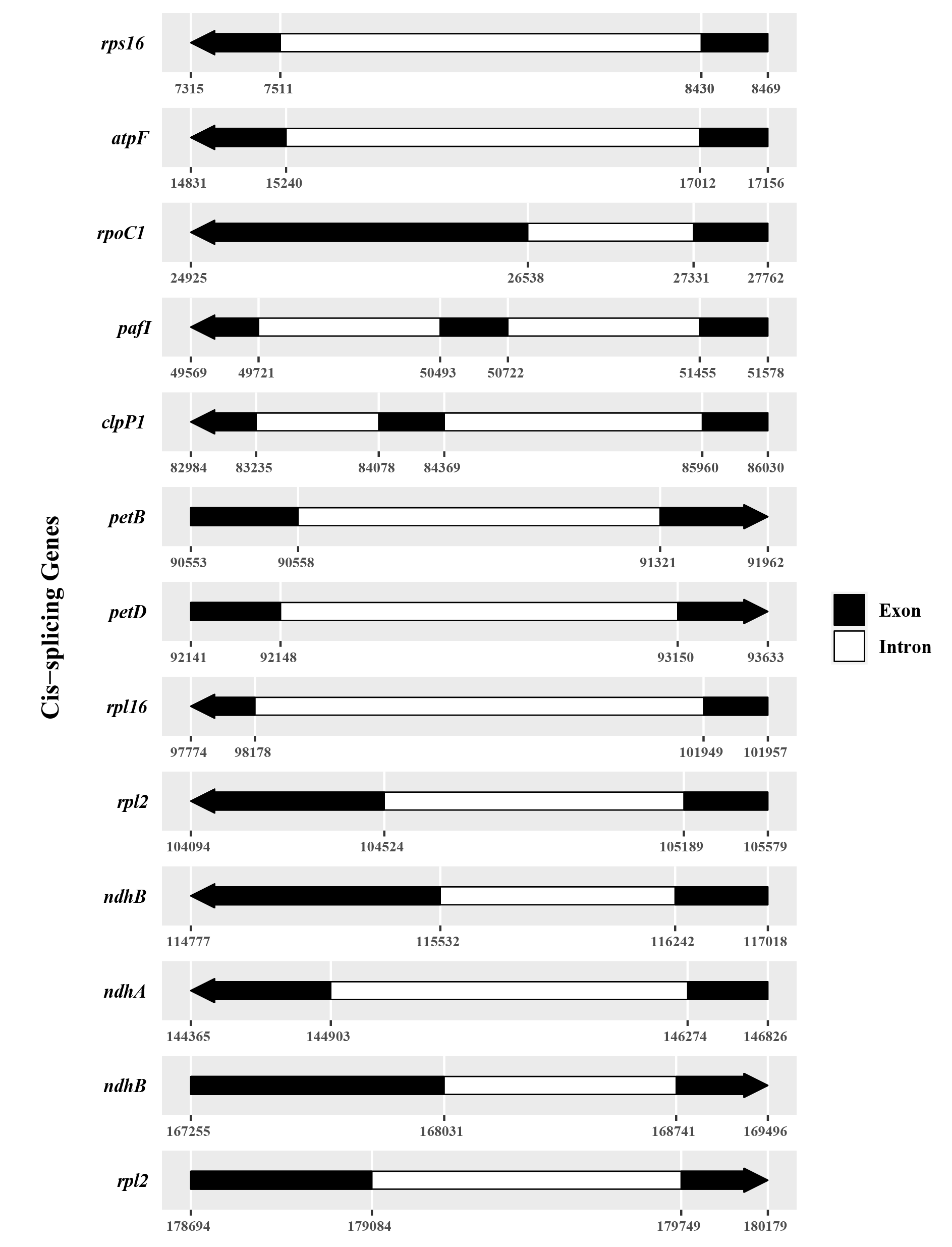

Supplement: Supplementary file 1 — Supplementary Figures [file 41598_2025_98287_MOESM1_ESM.zip › Figure S1.tif]

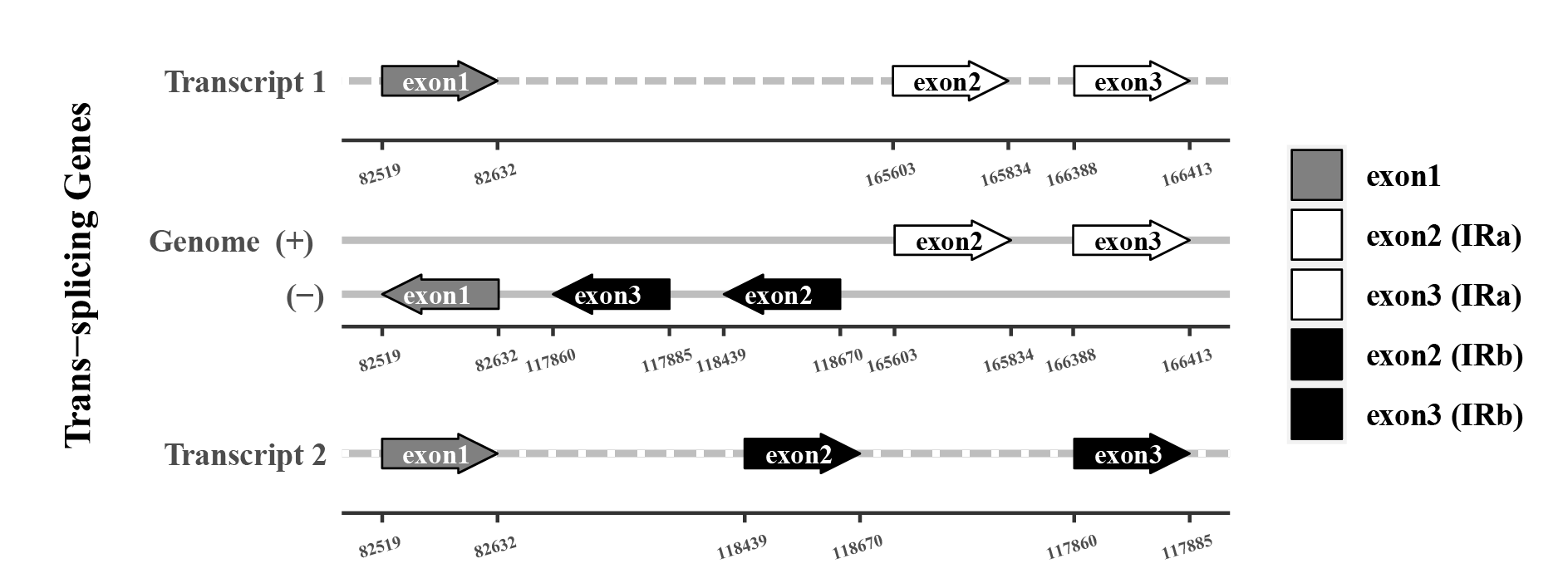

Supplement: Supplementary file 1 — Supplementary Figures [file 41598_2025_98287_MOESM1_ESM.zip › Figure S2.tif]

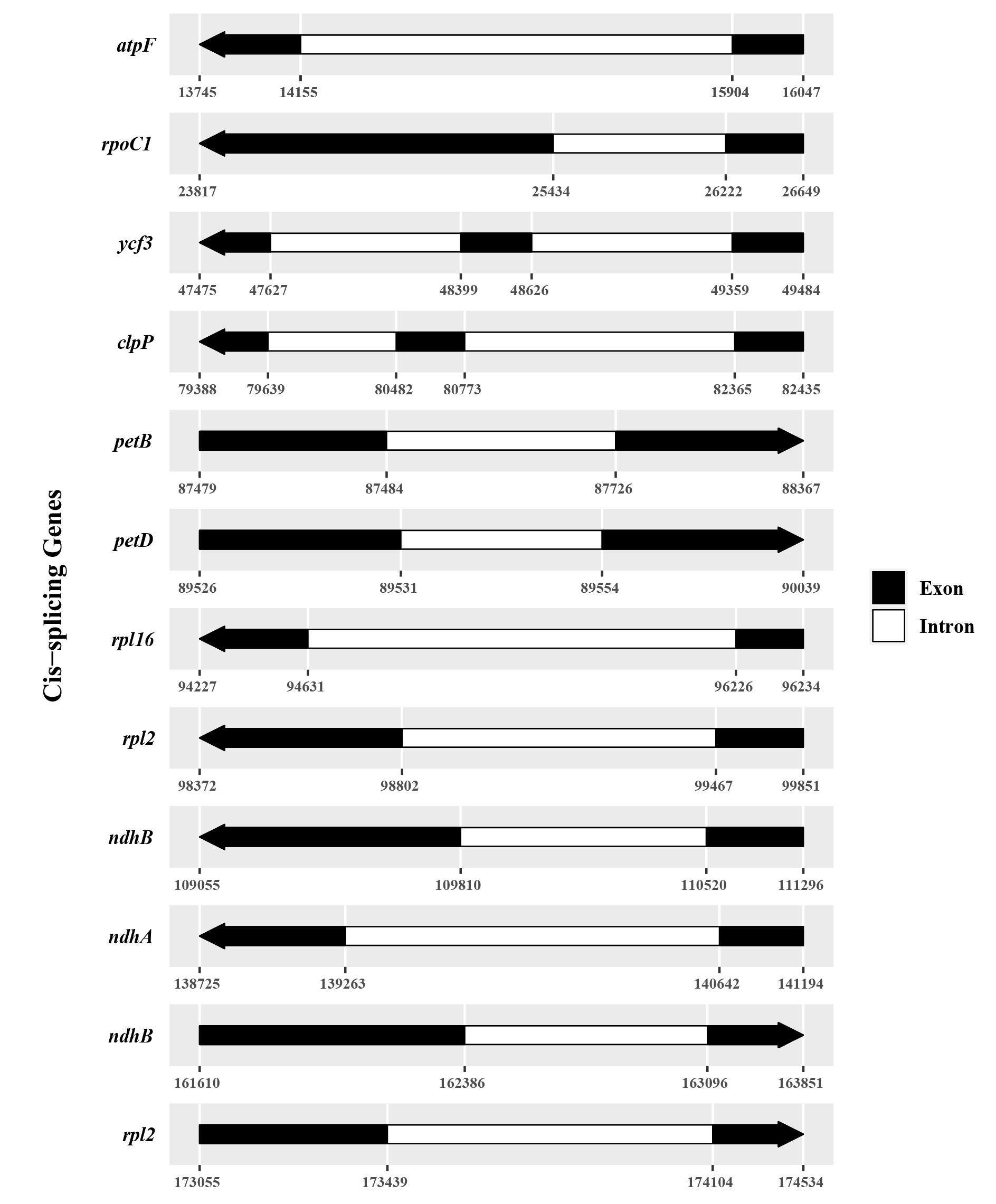

Supplement: Supplementary file 1 — Supplementary Figures [file 41598_2025_98287_MOESM1_ESM.zip › Figure S3.tif]

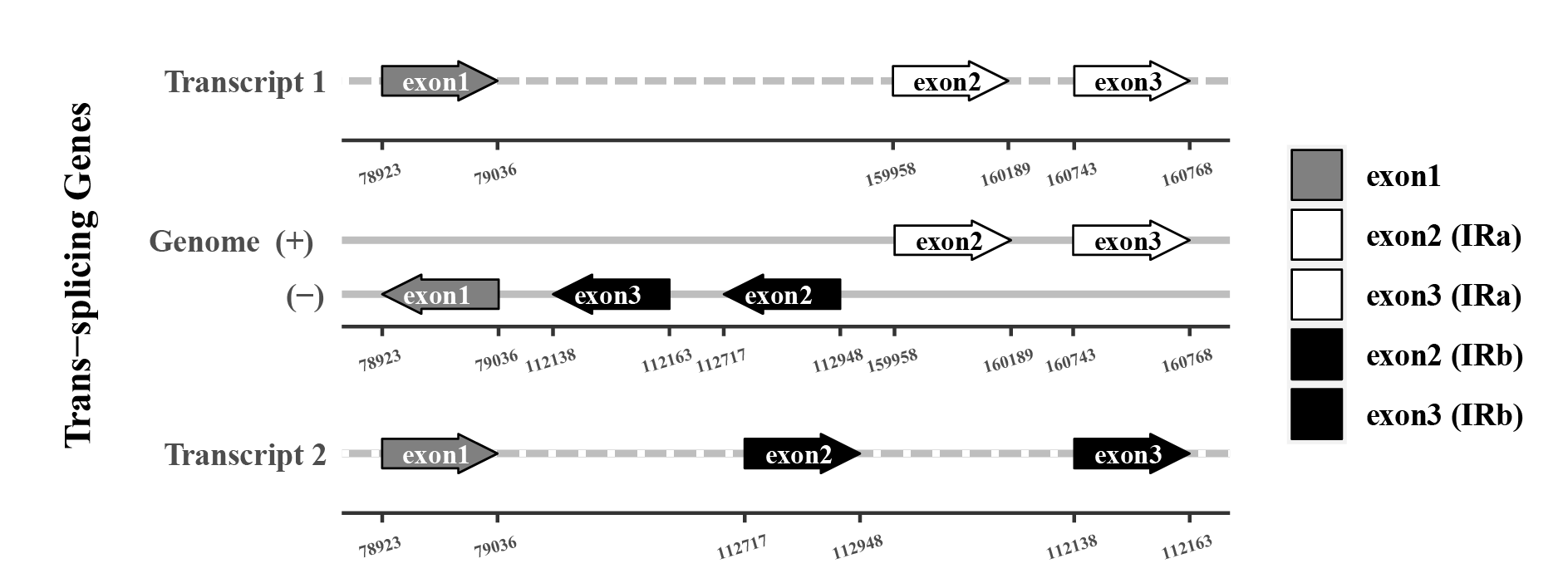

Supplement: Supplementary file 1 — Supplementary Figures [file 41598_2025_98287_MOESM1_ESM.zip › Figure S4.tif]

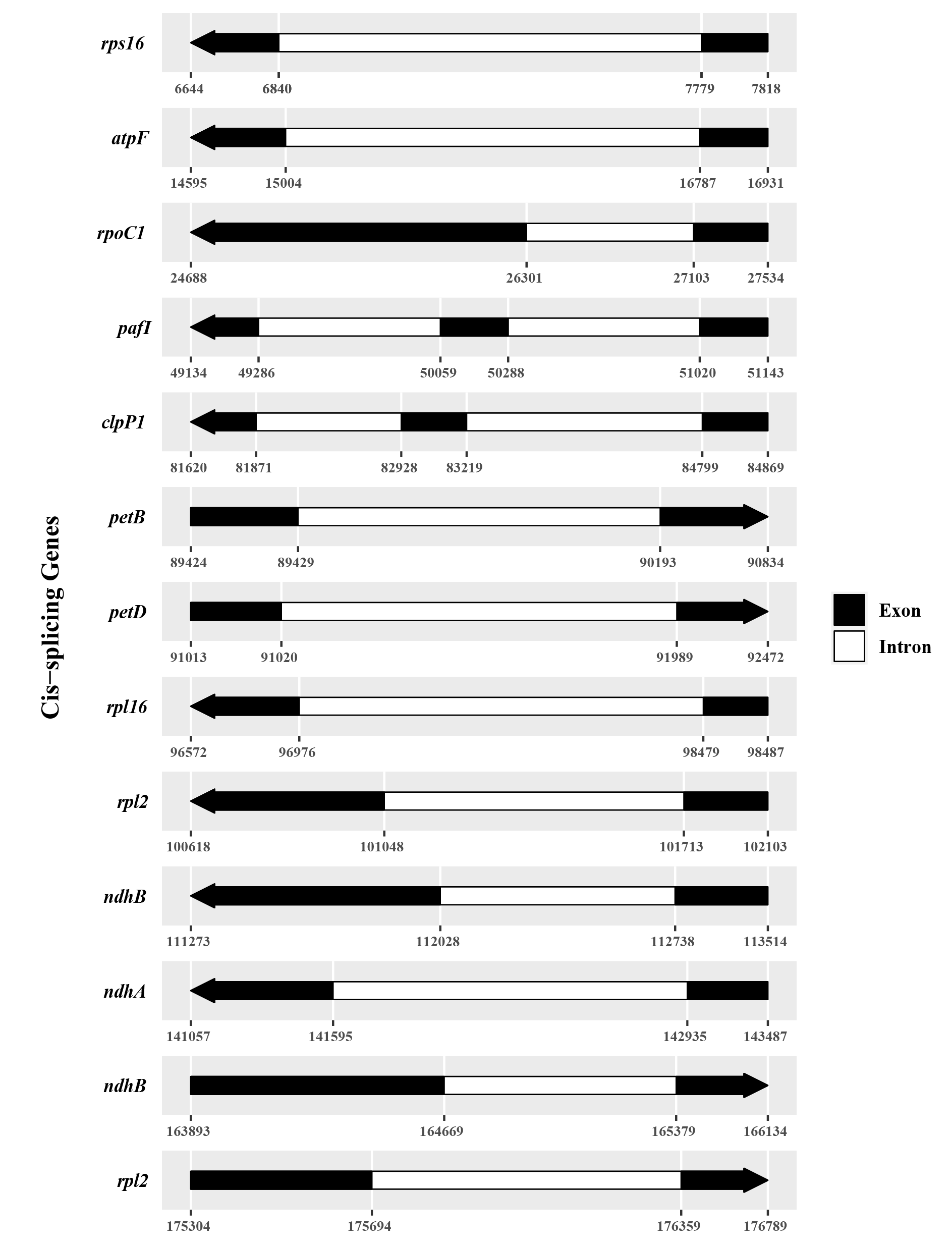

Supplement: Supplementary file 1 — Supplementary Figures [file 41598_2025_98287_MOESM1_ESM.zip › Figure S5.tif]

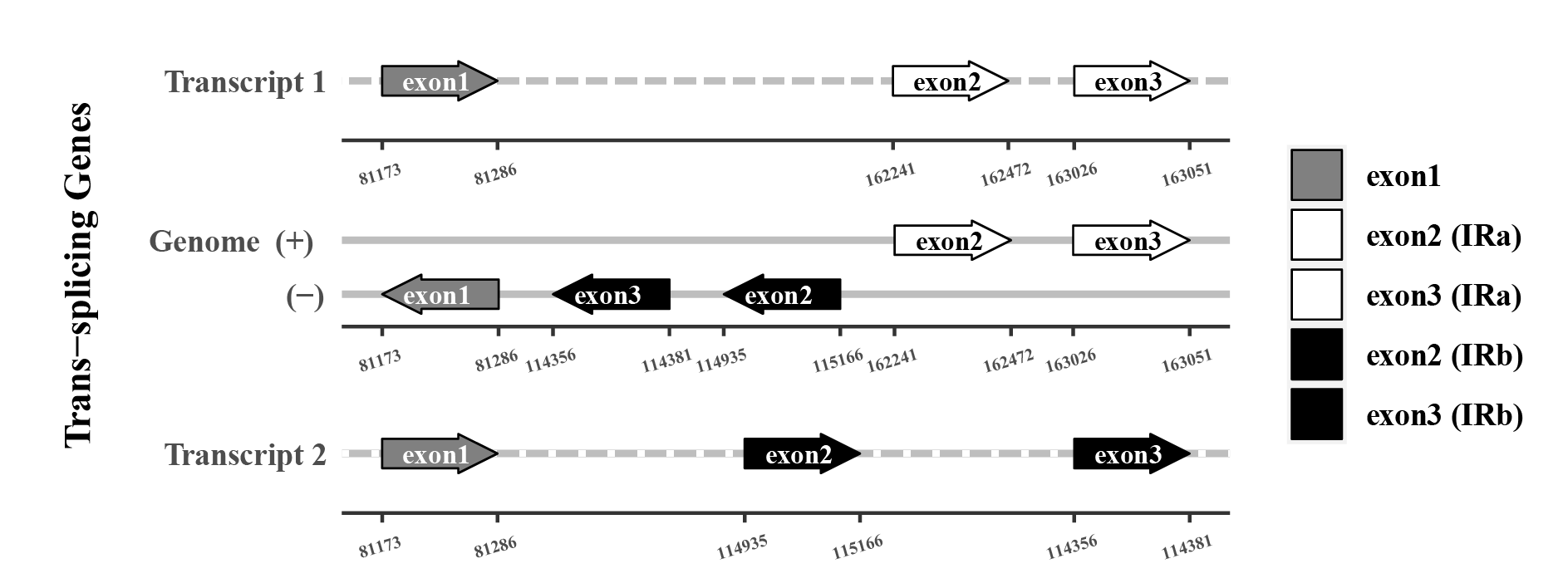

Supplement: Supplementary file 1 — Supplementary Figures [file 41598_2025_98287_MOESM1_ESM.zip › Figure S6.tif]

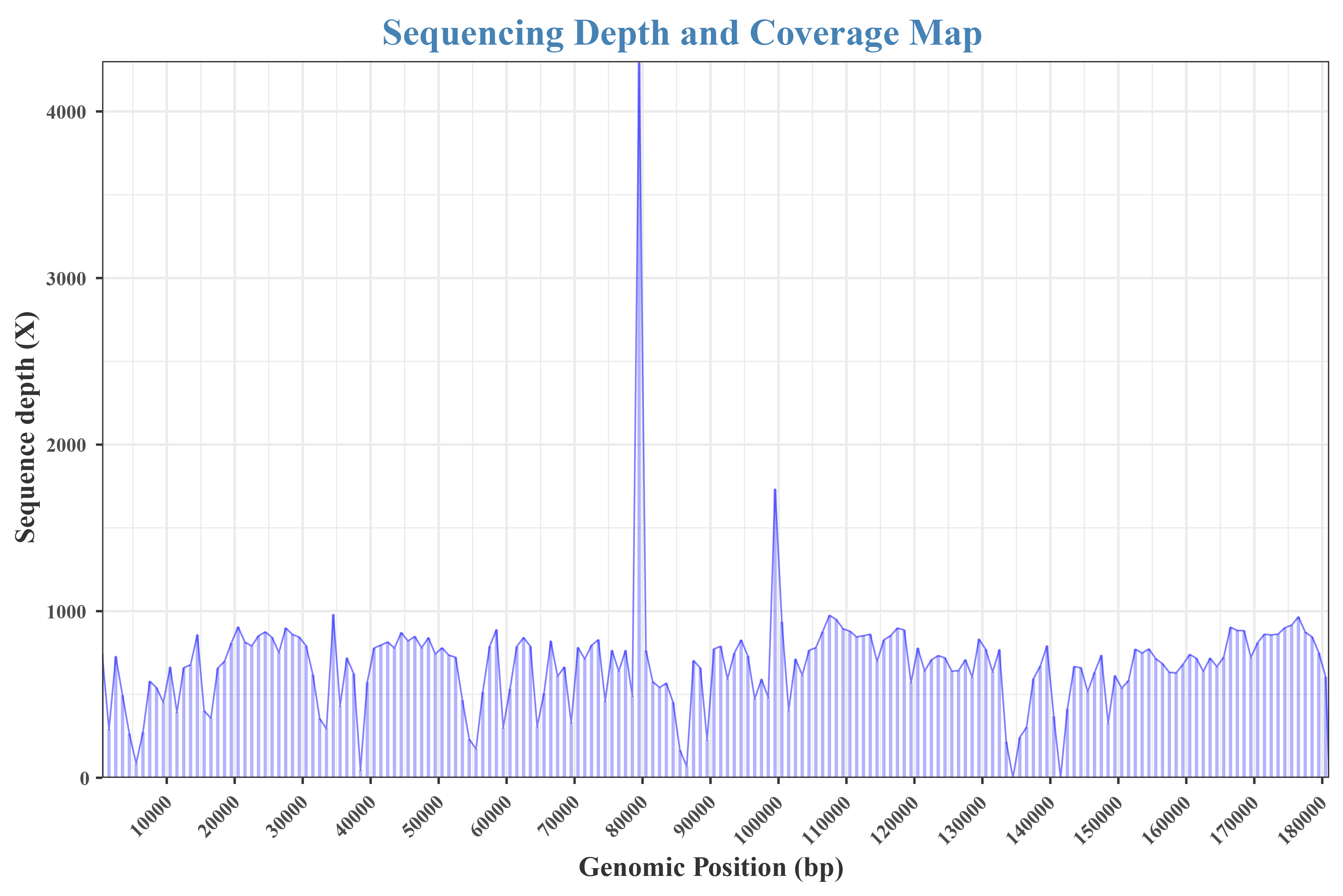

Supplement: Supplementary file 1 — Supplementary Figures [file 41598_2025_98287_MOESM1_ESM.zip › Figure S7.tif]

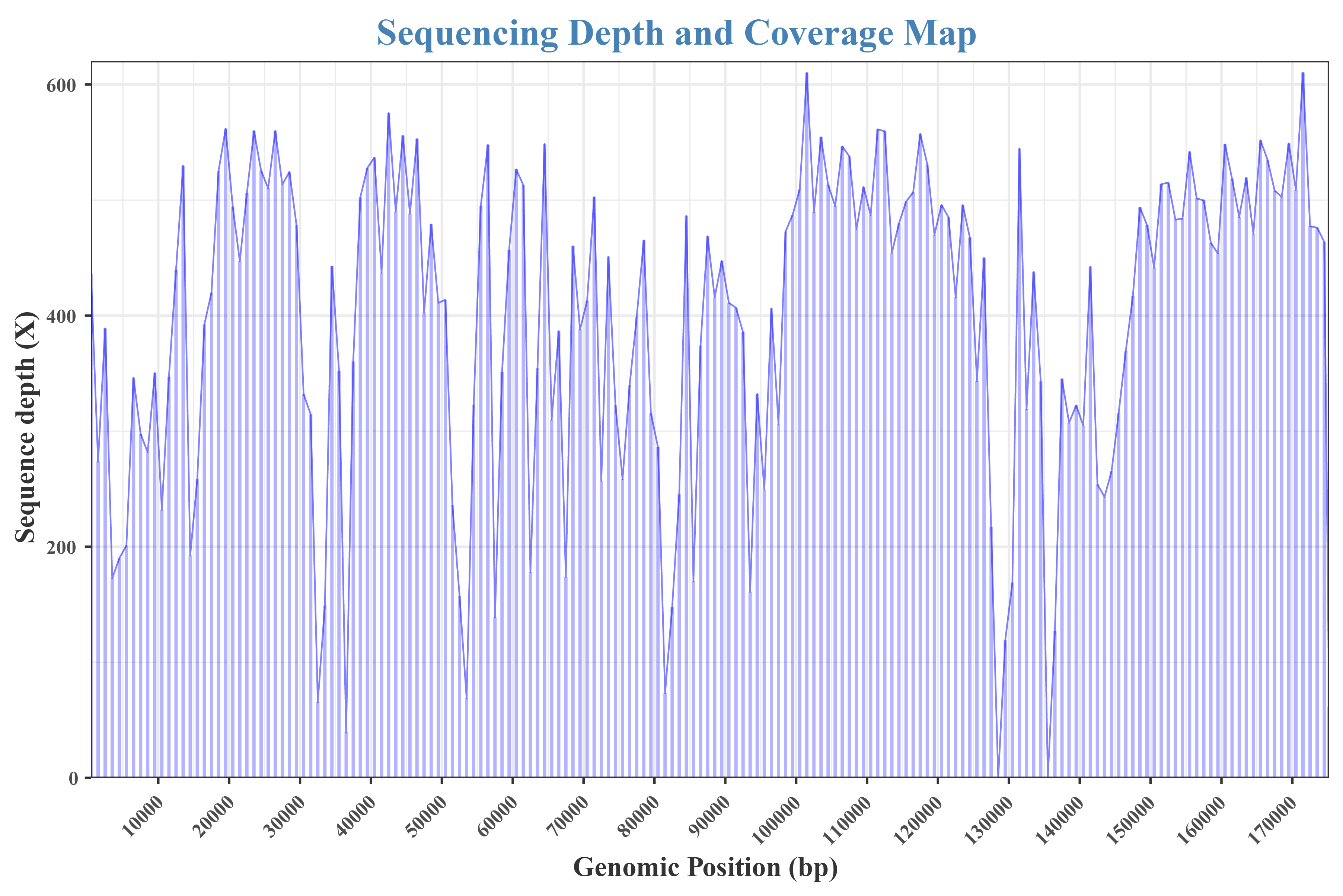

Supplement: Supplementary file 1 — Supplementary Figures [file 41598_2025_98287_MOESM1_ESM.zip › Figure S8.tif]

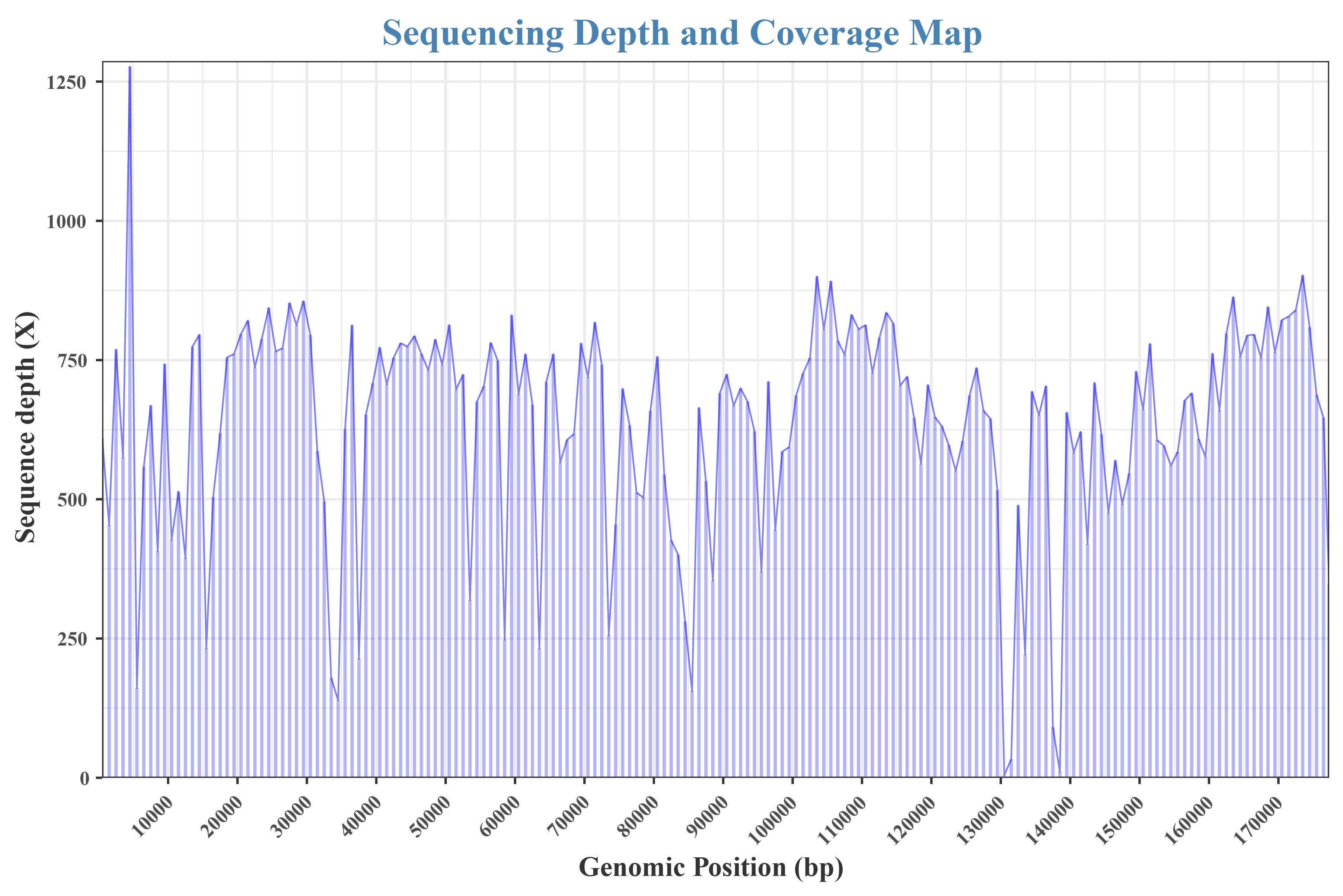

Supplement: Supplementary file 1 — Supplementary Figures [file 41598_2025_98287_MOESM1_ESM.zip › Figure S9.tif]
